# Cumulative incidence and survival outcomes of brain metastases in sarcoma: a large single center retrospective analysis

**DOI:** 10.1038/s41416-025-03111-4

**Published:** 2025-07-26

**Authors:** Ayah Erjan, Kurl Jamora, Enrique Gutierrez, Anna Santiago, Adeodatus Vito Nicanor, Barbara-Ann Millar, Normand Laperriere, Tatiana Conrad, Dana Keilty, Philip Wong, Peter Chung, Charles Catton, David Kirsch, David B. Shultz

**Affiliations:** 1 Department of Radiation Oncology, University of Toronto, Toronto, ON Canada; 2 Radiation Medicine Program, Princess Margaret Cancer Centre, University Health Network, Toronto, On Canada; 3 Department of Radiation Oncology, King Hussein Cancer Centre, Amman, Jordan; 4 Department of Biostatistics, University of Toronto, Toronto, On Canada

**Keywords:** Sarcoma, CNS cancer

## Abstract

**Background:**

The incidence and predictors of brain metastases (BrM) from sarcoma remain poorly characterized. We aimed to determine the cumulative incidence (CuI) and risk factors for BrM.

**Methods:**

We retrospectively analyzed data from all sarcoma patients who presented to our center (2006–2023). CuI was calculated from initial presentation to BrM, stratified by key variables. Univariable (UVA) and multivariable competing risk regression analyses (MVA) were conducted to identify risk factors.

**Results:**

Among 5110 sarcoma patients, 117 developed BrM. CuI rates were 1.8%, 2.4%, and 2.9% at 24, 48, and 72 months, respectively, within a median onset of 17 months. On UVA, intrathoracic primary site, alveolar soft part (ASPS), epithelioid, intimal and Rhabdomyosarcoma histologies, and stage IV at diagnosis were associated with increased CuI, while age ≥59, retroperitoneal origin and liposarcoma were associated with decreased CuI. On MVA the following remained correlated to BrM incidence: intrathoracic primary (HR 5.13), ASPS (HR 4.2), age ≥59 years (HR 0.45) and liposarcoma (HR 0.11); 44.3% presented with solitary BrM. Median survival post-BrM diagnosis was 6 months.

**Conclusion:**

BrM risk in sarcoma varies by age, histology, and tumor location. Solitary metastases were common in our BrM cohort, and OS post-BrM was poor.

## Introduction

Brain metastases (BrM) from carcinomas are relatively common, occurring in up to 50% of patients [1]. However, for reasons that are totally unknown, sarcomas, a diverse group of mesenchymal cancers, rarely metastasize to brain [1–5].

The incidence and risk factors for BrM in sarcoma patients remain unclear, due to their rarity and the extensive heterogeneity within sarcoma histologies. BrM incidence has been reported to be associated with primary tumor location and histological subtype [1, 6]. However, that data is mostly derived from population-based registries that only record BrM diagnosed at sarcoma presentation or from small to moderate sized retrospective series.

We aimed to calculate the cumulative incidence and clinical factors associated with BrM in adult sarcoma patients through analysis of data from a large cohort of patients treated at our tertiary cancer center. Our goal was to identify highest-risk patient phenotypes.

## Methods

### Study design and population

Patients aged 18 and older, diagnosed with pathologically-confirmed bone or soft tissue sarcoma (STS) were eligible for inclusion. We excluded BrM patients with multiple primary tumors, patients with primary brain sarcomas, or metastases arising from the skull with brain invasion. After obtaining approval from the University Health Network’s (UHN) Research Ethics Board, we accessed the records of patients diagnosed with bone or soft tissue sarcomas between January 1, 2006, and January 1, 2023. In addition, 53 additional patients diagnosed before 2006, who presented to our center with recurrent disease following initial treatment elsewhere, were included in the study. Follow-up extended until January 1, 2024. Patients who were initially diagnosed outside Princess Margaret Cancer Center (PMCC) were eligible provided their sarcoma pathology was reviewed and confirmed at our center.

### Data collection

Comprehensive data extraction from patient charts included age at primary sarcoma diagnosis, gender, primary tumor diagnosis date, primary tumor location, histologic subtype, tumor origin (bone vs. soft tissue), stage at diagnosis (localized vs. metastatic), last follow-up date, and status at last follow-up. Cases of BrM were identified from cross-checking our prospective BrM and radiation therapy databases (Mosaiq). The date of the first magnetic resonance image (MRI) confirming parenchymal or dural-based BrM diagnosis was used as the BrM diagnosis date. BrM were primarily identified through imaging prompted by the onset of neurological symptoms. Routine screening MRIs were not performed, and no standardized protocol for brain imaging was applied across the cohort.

### Statistical analysis

#### Cumulative incidence (CuI)

CuI was measured in months, starting from sarcoma diagnosis at up to 3 potential points in time: a) date of BrM diagnosis or b) date of last follow-up if the patient did not develop BrM and was alive at last follow-up (Censored), or c) date of death if the patient did not develop BrM (competing risk event).

#### Descriptive statistics

Variables were classified and summarized as follows: categorical variables were presented as frequencies and percentages, while continuous variables were represented as medians along with their ranges and interquartile ranges (IQR).

#### Comparative analysis

We employed the Wilcoxon rank-sum test for continuous variables, and either Fisher’s exact test or Chi-squared test for categorical variables to compare patient characteristics between those who developed BrM and those who did not.

#### Time-to-event analysis

The time from sarcoma diagnosis to the onset of BrM was analyzed using the CuI function, considering death as a competing risk. CuI curves were assessed across different demographic and clinical characteristics using Gray’s test.

#### Regression analysis

Univariable and multivariable competing risk regression analyses were conducted using the Fine and Gray method to estimate the subdistribution hazard ratios (HR) for factors associated with BrM development. Adjusted subdistribution HR were calculated for the variables that showed significant associations.

#### Overall survival (OS) analysis

For patients who developed BrM, OS was calculated using the Kaplan–Meier method. OS was defined from the date of BrM development until death or last follow-up.

All statistical analyses were performed using R version 4.3.1 (R Core Team, 2023).

## Results

A total of 5110 patients were enrolled to the study, among whom 117 presented with BrM either at initial presentation or during their follow-up. The baseline characteristics of the entire cohort are presented in Table 1. Median age at diagnosis was 59 years, (range, 18–98 years), with a slight male predominance identified (*n* = 2706, 53.0%). Most patients (90.5% of the cohort) had STS (*n* = 4624).

**Table 1:** Patient characteristics.

All sarcoma patients by brain metastasis yes/no				
	Full sample (*n* = 5110)	Yes (*n* = 117)	No (*n* = 4993)	*p*-value
Gender				0.93
Female	2404 (47.0)	56 (47.9)	2348 (47.0)	
Male	2706 (53.0)	61 (52.1)	2645 (53.0)	
Age diagnosis				**<0.001**
Mean (sd)	57.5 (17.5)	45.8 (16.1)	57.8 (17.4)	
Median (Min,Max)	59 (18, 98)	47 (18, 80)	59 (18, 98)	
Age diagnosis group				**<0.001**
<median of 59 years	2528 (49.5)	87 (74.4)	2441 (48.9)	
59 years and above	2582 (50.5)	30 (25.6)	2552 (51.1)	
Location primary				**<0.001**
Extremities	1408 (27.6)	38 (32.5)	1370 (27.4)	
Trunk	982 (19.2)	24 (20.5)	958 (19.2)	
H&N	473 (9.3)	13 (11.1)	460 (9.2)	
Retroperitoneal	686 (13.4)	8 (6.8)	678 (13.6)	
Intrathoracic	131 (2.6)	16 (13.7)	115 (2.3)	
Uterine	431 (8.4)	16 (13.7)	415 (8.3)	
Pelvic (non-uterine)	52 (1.0)	1 (0.9)	51 (1.0)	
Liver	34 (0.7)	1 (0.9)	33 (0.7)	
Others	434 (8.5)	0 (0.0)	434 (8.7)	
Unknown	479 (9.4)	0 (0.0)	479 (9.6)	
Histologic subtype				**<0.001**
UPS	798 (15.6)	24 (20.5)	774 (15.5)	
Angiosarcoma	250 (4.9)	6 (5.1)	244 (4.9)	
ASPS	20 (0.4)	7 (6.0)	13 (0.3)	
Chondrosarcoma	180 (3.5)	0 (0.0)	180 (3.6)	
Clear cell sarcoma	20 (0.4)	1 (0.9)	19 (0.4)	
Endometrial stromal sarcoma (ESS)	83 (1.6)	1 (0.9)	82 (1.6)	
Epithelioid hemangioendothelioma (EHE)	29 (0.6)	1 (0.9)	28 (0.6)	
Epithelioid sarcoma	62 (1.2)	5 (4.3)	57 (1.1)	
Ewing sarcoma	89 (1.7)	4 (3.4)	85 (1.7)	
Fibrosarcoma	59 (1.2)	2 (1.7)	57 (1.1)	
Intimal sarcoma	13 (0.3)	3 (2.6)	10 (0.2)	
Leiomyosarcoma	840 (16.4)	28 (23.9)	812 (16.3)	
Liposarcoma	742 (14.5)	2 (1.7)	740 (14.8)	
MPNST	121 (2.4)	3 (2.6)	118 (2.4)	
Myxofibrosarcoma	261 (5.1)	5 (4.3)	256 (5.1)	
Myxoid chondrosarcoma	29 (0.6)	1 (0.9)	28 (0.6)	
Myxoid liposarcoma	143 (2.8)	1 (0.9)	142 (2.8)	
Osteosarcoma	175 (3.4)	8 (6.8)	167 (3.3)	
Others	899 (17.6)	0 (0.0)	899 (18.0)	
Rhabdomyosarcoma	116 (2.3)	9 (7.7)	107 (2.1)	
Synovial sarcoma	181 (3.5)	6 (5.1)	175 (3.5)	
Origin primary				0.91
Bone	486 (9.5)	12 (10.3)	474 (9.5)	
Soft tissue	4624 (90.5)	105 (89.7)	4519 (90.5)	
Stage				**<0.001**
Localized	3579 (80.0)	79 (67.5)	3500 (80.4)	
Metastatic	892 (20.0)	38 (32.5)	854 (19.6)	
Missing	639	0	639	

Statistically significant *p* < 0.05 values are in bold.

We grouped patients into more than 20 histologies, including leiomyosarcoma, undifferentiated pleomorphic sarcoma (UPS) and liposarcoma, which accounted for 16.4% (*n* = 840), 15.6%, *n* = 798), and 14.5%, *n* = 742) of cases, respectively. Rarer subtypes that did not develop BrM throughout the study period were categorized under ‘Others,’ comprising 17.6% (*n* = 899) of the entire cohort, the most common entities in this group were Gastrointestinal Stromal tumors (GIST), solitary fibrous tumor and Kaposi’s sarcoma.

Primary sarcoma location was available in 90.6% patients (*n* = 4631). Most tumors arose in the extremities 27.6% (*n* = 1408), followed by the trunk 19.2% (*n* = 982), retroperitoneum 13.4% (*n* = 686) and head and neck 9.3% (*n* = 473). Other sites are detailed in Table 1. For classification purposes, ‘intrathoracic’ included tumors originating in the lung, mediastinum, pleura or cardiac regions, while ‘trunk’ referred to sarcomas of the soft tissue in the chest wall, back or abdominal wall. Stage data was available in 87.5% patients (*n* = 4471). At the time of diagnosis, 80.0% of patients (*n* = 3579) had localized disease, while 20.0% (*n* = 892) presented with metastatic disease.

### Cumulative incidence of BrM

**Fig. 1:**
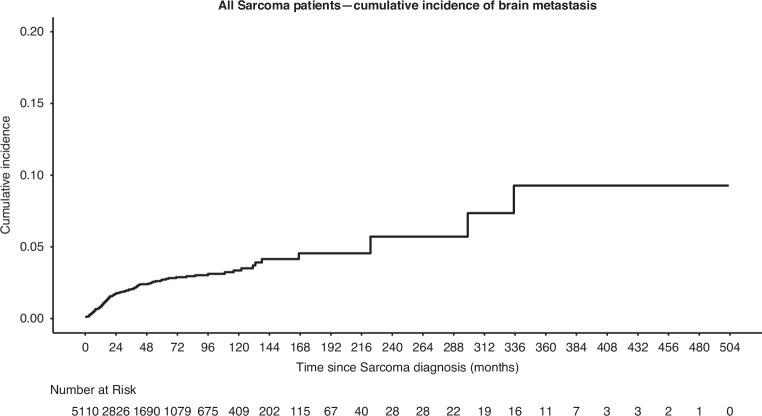
Cumulative incidence of brain metastases (BrM). Curve shows BrM incidence from sarcoma diagnosis; number at risk is shown below.

**Fig. 2:**
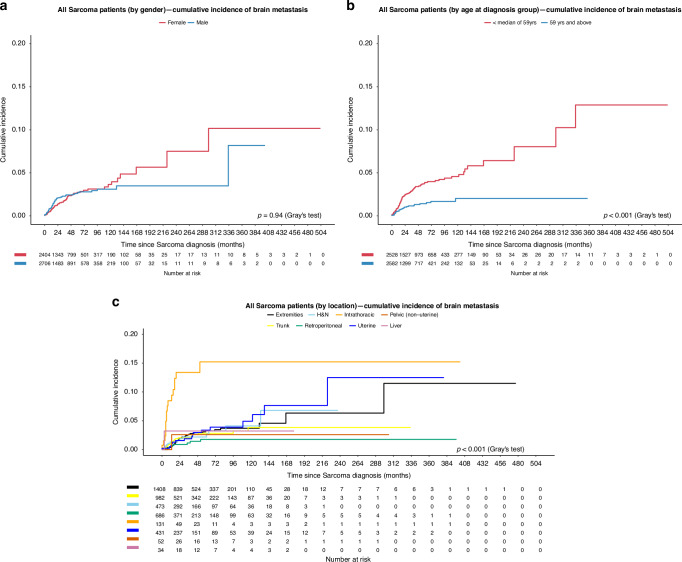
Cumulative incidence of brain metastases by gender, age at diagnosis and location of the primary sarcoma. Panels show CuI curves stratified by (**a**) gender, (**b**) age group (59 vs. ≥59 years), and (**c**) sarcoma location; Gray’s test pvalues shown.

**Fig. 3:**
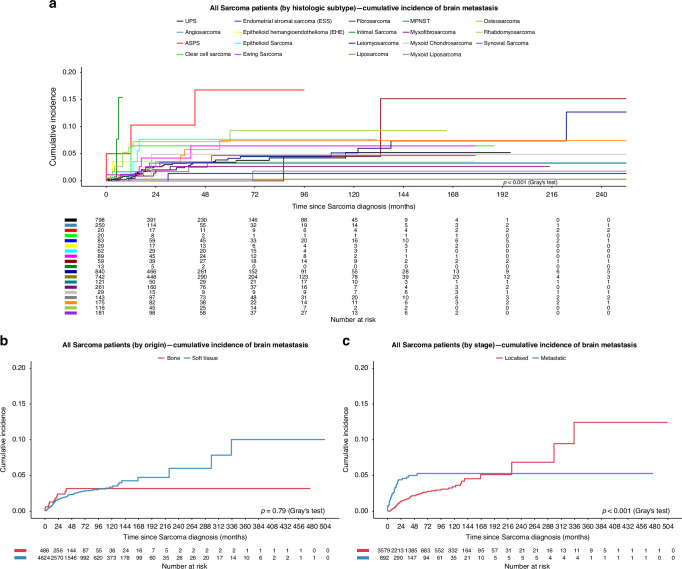
Cumulative incidence of brain metastases by histologic subtype, primary sarcoma origin and stage at diagnosis. Panels show CuI by (**a**) histology, (**b**) bone vs. soft tissue origin, and (**c**) localized vs. metastatic stage; number at risk displayed; *p*-values from Gray’s test.

CuI rates for the entire cohort were 1.8% (95% CI [0.014, 0.022]), 2.4% (95% CI [0.019, 0.029]), and 2.9% (95% CI [0.023, 0.035]) at 24, 48, and 72 months, respectively, with a median Time To BrM (TTBrM) of 17 months (range, 0-335), and mean TTBrM 33.7 months (SD, 52.3) (Fig. 1). CuI curves stratified by gender, age, primary location, histological subtypes, sarcoma origin, and stage are represented in Figs. 2 and 3. Although the female CuI curve appears higher at later timepoints, males had a higher 24-month incidence (2.1% vs. 1.4%), and this difference was not statistically significant (Gray’s test, *p* = 0.94) Fig. 2.

### Characteristics and outcomes of patients who developed brain metastases

**Fig. 4:**
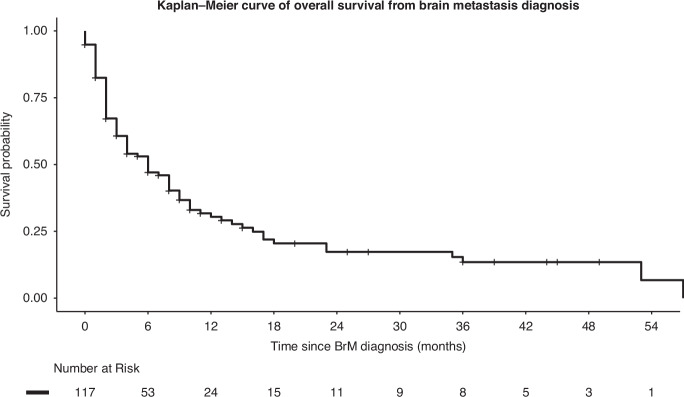
Kaplan–Meier curve of overall survival from brain metastases diagnosis. Survival probability over time following BrM diagnosis; tick marks represent censored observations.

Among 117 patients who developed BrM during the study, median survival post-BrM diagnosis was 6 months (95% CI [4, 9]). Overall survival (OS) rates at 6, 12, and 18 months were 47.1% (95% CI [38.5%, 57.7%]), 30.4% (95% CI [22.4%, 41.4%]), and 20.5% (95% CI [13.4%, 31.4%]), respectively (Fig. 4). The median age at diagnosis was 47 years (range: 18–80). Fifty-five percent were males (*n* = 56).

Histologically, leiomyosarcoma was predominant (28/117 cases, 23.9%), followed by UPS (20.5%, *n* = 24), Rhabdomyosarcoma (7.7%, *n* = 9), Osteosarcoma (6.8%, *n* = 8) and Alveolar Soft Part Sarcoma (ASPS) (6.0%, *n* = 7). The majority (89.7%, *n* = 105) of BrM originated from STS as opposed to bone sarcomas.

The most common primary site was in an extremity (32.6%, *n* = 38), followed by the trunk (20.5%, *n* = 24), and intra-thoracic or uterine (13.7%%, *n* = 16, each). Notably, in 24 patients the brain was the first identified site of metastasis, whereas the majority of BrM were preceded by visceral metastases, predominantly in the lungs.

Most patients presented with a single metastasis (44.3%, *n* = 51), 25.2% had 2–4 metastases, and the remainder with multiple BrM; 39.7% (*n* = 46) of patients had at least 1 hemorrhagic BrM. Sixty-four percent of patients had solely supratentorial BrM, 12% were infratentorial and 25% had both supra and infratentorial BrM. Ten percent of BrM patients developed leptomeningeal carcinomatosis.

### Treatment modalities for BrM

Among the 117 patients with BrM, all received treatment except for two patients who were ineligible due to extremely poor performance status. The remaining 115 patients received surgery, stereotactic radiosurgery (SRS), whole-brain radiotherapy (WBRT), or a combination of these modalities (Table 2). Radiotherapy was the most commonly administered treatment. Specifically, 38 patients (33.0%) received WBRT, 35 (30.4%) received SRS, 4 (3.5%) received WBRT with an SRS boost, and 36 (30.8%) received radiotherapy as adjuvant therapy following surgery. Median survival by treatment strategy is summarized in Table 3. Patients who received surgery followed by adjuvant SRS had the longest observed median survival of 14 months, whereas those who received WBRT alone had the shortest median survival of 2 months. Additionally, patients who received surgery followed by SRS achieved 18 months OS of 34.5% (95% CI: 17.2–69.2%).

**Table 2:** Distribution of treatment modalities among patients with brain metastases, stratified by survival status, including mutually exclusive therapy groups.

	Full sample (*n* = 117)	Alive (*n* = 32)	Dead (*n* = 85)
SRS Yes 0 No 1			
No 1	**53 (46.1)**	11 (34.4)	42 (50.6)
Yes 0	**62 (53.9)**	21 (65.6)	41 (49.4)
Missing	**2**	0	2
Surgey Yes 0 No 1			
No 1	**77 (67.0)**	19 (59.4)	58 (69.9)
Yes 0	**38 (33.0)**	13 (40.6)	25 (30.1)
Missing	**2**	0	2
WBRT Yes 0 No 1			
No 1	**60 (52.2)**	20 (62.5)	40 (48.2)
Yes 0	**55 (47.8)**	12 (37.5)	43 (51.8)
Missing	**2**	0	2
Radiotherapy WBRT or SRS Yes 0 No 1			
No 1	**2 (1.7)**	0 (0.0)	2 (2.4)
Yes 0	**113 (98.3)**	32 (100.0)	81 (97.6)
Missing	**2**	0	2
Both surgery and RT Yes 0 No 1			
No 1	**78 (67.8)**	19 (59.4)	59 (71.1)
Yes 0	**37 (32.2)**	13 (40.6)	24 (28.9)
Missing	**2**	0	2
BrM therapies mutually exclusive			
SRS	**35 (30.4)**	11 (34.4)	24 (28.9)
WBRT	**38 (33.0)**	8 (25.0)	30 (36.1)
SRS and WBRT	**4 (3.5)**	0 (0.0)	4 (4.8)
Surgery	**2 (1.7)**	0 (0.0)	2 (2.4)
Surgery and SRS	**22 (19.1)**	9 (28.1)	13 (15.7)
Surgery and WBRT	**13 (11.3)**	3 (9.4)	10 (12.0)
Surgery with SRS and WBRT	**1 (0.9)**	1 (3.1)	0 (0.0)
Missing	**2**	0	2

Statistically significant *p* < 0.05 values are in bold.

**Table 3:** Median overall survival and 6-, 12-, and 18-month survival probabilities by mutually exclusive treatment groups in patients with brain metastases.

Group	Events/Total	Median (95% CI)	6months (95% CI)	12months (95% CI)	18months (95% CI)
SRS	24/35	**8.0 (6.0, 23.0)**	0.528 (0.383, 0.727)	0.297 (0.168, 0.524)	0.297 (0.168, 0.524)
WBRT	30/38	**2.0 (1.0, 4.0)**	0.255 (0.140, 0.466)	0.170 (0.074, 0.389)	0.043 (6.44e-03, 0.281)
SRS and WBRT	4/4	**9.5 (2.0, NA)**	0.750 (0.426, 1.000)	0.00 (NA, NA)	0.00 (NA, NA)
Surgery	2/2	**12.5 (2.0, NA)**	0.500 (0.125, 1.000)	0.500 (0.125, 1.000)	0.500 (0.125, 1.000)
Surgery and SRS	13/22	**14.0 (7.0, NA)**	0.679 (0.508, 0.907)	0.503 (0.321, 0.787)	0.345 (0.172, 0.692)
Surgery and WBRT	10/13	**6.0 (4.0, NA)**	0.463 (0.245, 0.875)	0.463 (0.245, 0.875)	0.231 (0.072, 0.745)
Surgery with SRS and WBRT	0/1	**NA (NA, NA)**	1.000 (1.000, 1.000)	1.000 (1.000, 1.000)	1.000 (1.000, 1.000)
			Log Rank Test	ChiSq	19.5 on 6 df
				***p*-value**	**0.003**

Statistically significant *p* < 0.05 values are in bold.

*NA* not reached - Surgery with SRS and WBRT (no death at last follow-up).

### Univariable and multivariable competing risk regression analysis

According to univariable analyses (UVA), several factors associated with an increased risk of BrM. Intrathoracic primary location (HR 4.96, 95% CI [2.72, 9.06], *P* < 0.001), alveolar soft part sarcoma (ASPS) (HR 7.22, 95% CI [3.5, 14.9], *P* < 0.001), epithelioid sarcoma (HR 3.11, 95% CI [1.19, 8.09] *P* = 0.02), intimal sarcoma(HR 8.58, 95% CI [2.39, 30.8], *P* < 0.001), rhabdomyosarcoma (HR 2.62, 95% CI [1.21, 5.7], *P* = 0.015) and metastatic stage at diagnosis (HR 2.08, 95% CI [1.41, 3.06], *P* < 0.001) were positively correlated to BrM incidence (Table 4). Factors associated with a decreased risk of BrM included age ≥59 years (HR 0.38, 95% CI [0.25, 0.58], *P* < 0.001), primary retroperitoneal origin (HR 0.40, 95% CI [0.18, 0.85], *P* = 0.018), and liposarcomas (HR 0.09, 95% CI [0.02, 0.37], *P* < 0.001) (Table 4).

**Table 4:** Univariable competing risk regression.

Variable	HR	95% CI	*p*-value
Gender			
Female (reference)	–	–	
Male	1.10	0.77, 1.58	0.590
Age_Diagnosis_Group			
< median of 59 years (reference)	–	–	
59 years and above	0.38	0.25, 0.58	<0.001
Location_Primary			
Extremities (reference)	–	–	
Trunk	0.97	0.58, 1.61	0.890
H&N	1.13	0.61, 2.12	0.690
Retroperitoneal	0.40	0.18, 0.85	0.018
Intrathoracic	4.96	2.72, 9.06	<0.001
Uterine	1.08	0.61, 1.93	0.790
Pelvic (non-uterine)	1.23	0.17, 9.12	0.840
Liver	1.13	0.15, 8.55	0.910
Histologic_Subtype			
UPS (reference)	–	–	
Angiosarcoma	0.72	0.29, 1.77	0.480
ASPS	7.22	3.50, 14.9	<0.001
Clear cell sarcoma	1.45	0.19, 11.1	0.720
Endometrial stromal sarcoma (ESS)	0.27	0.04, 2.00	0.200
Epithelioid hemangioendothelioma (EHE)	1.03	0.13, 7.96	0.970
Epithelioid Sarcoma	3.11	1.19, 8.09	0.020
Ewing Sarcoma	1.38	0.48, 3.98	0.550
Fibrosarcoma	0.98	0.24, 3.94	0.970
Intimal Sarcoma	8.58	2.39, 30.8	<0.001
Leiomyosarcoma	1.00	0.58, 1.72	0.990
Liposarcoma	0.09	0.02, 0.37	<0.001
MPNST	0.84	0.25, 2.81	0.780
Myxofibrosarcoma	0.77	0.30, 2.03	0.600
Myxoid Chondrosarcoma	1.00	0.13, 7.56	>0.999
Myxoid Liposarcoma	0.22	0.03, 1.64	0.140
Osteosarcoma	1.40	0.63, 3.11	0.410
Rhabdomyosarcoma	2.62	1.21, 5.70	0.015
Synovial Sarcoma	1.13	0.46, 2.76	0.790
Origin_Primary			
Bone (reference)	–	–	
Soft tissue	0.73	0.40, 1.33	0.300
Stage			
Localized (reference)	–	–	
Metastatic	2.08	1.41, 3.06	<0.001

Excluded patients with Location-Unknown/Others, Histologic Subtype-Chondrosarcoma/ Others (no brain metastasis).

*SHR* subdistribution hazard ratio, Fine and Gray.

On MVA, the following predictors remained significantly associated with BrM risk: intrathoracic primaries (HR 5.13, 95% CI [2.47, 10.7], *P* < 0.001), ASPS histologic subtype (HR 4.2, 95% CI [1.83, 9.64], *P* < 0.001), liposarcomas (HR 0.11, 95% CI [0.02–0.53], *P* = 0.006) and age ≥59 years (HR 0.45, 95% CI [0.29, 0.71], *P* < 0.001) (Table 5).

**Table 5:** Multivariable competing risk regression for brain metastases in sarcoma.

Multivariable competing risk regression for brain metastasis in sarcoma			
Variable	HR	95% CI	*p*-value
Age_Diagnosis_Group			
< median of 59 years	–	–	
59 years and above	0.45	0.29, 0.71	<0.001
Location_Primary			
Extremities	–	–	
Trunk	1.05	0.62, 1.77	0.860
H&N	1.33	0.70, 2.52	0.380
Retroperitoneal	0.73	0.33, 1.62	0.430
Intrathoracic	5.13	2.47, 10.7	<0.001
Uterine	0.91	0.45, 1.85	0.800
Pelvic (non-uterine)	0.83	0.12, 5.83	0.850
Liver	1.82	0.38, 8.67	0.450
Histologic_Subtype			
UPS	–	–	
Angiosarcoma	0.82	0.28, 2.42	0.720
ASPS	4.20	1.83, 9.64	<0.001
Clear cell sarcoma	1.42	0.19, 10.8	0.730
Endometrial stromal sarcoma (ESS)	0.23	0.03, 1.88	0.170
Epithelioid hemangioendothelioma (EHE)	0.28	0.05, 1.45	0.130
Epithelioid Sarcoma	2.47	0.92, 6.63	0.072
Ewing Sarcoma	0.85	0.28, 2.59	0.780
Fibrosarcoma	0.78	0.19, 3.21	0.730
Intimal Sarcoma	2.09	0.50, 8.77	0.320
Leiomyosarcoma	1.02	0.55, 1.87	0.960
Liposarcoma	0.11	0.02, 0.53	0.006
MPNST	0.71	0.20, 2.49	0.590
Myxofibrosarcoma	0.83	0.32, 2.18	0.710
Myxoid Chondrosarcoma	0.77	0.10, 6.13	0.810
Myxoid Liposarcoma	0.18	0.02, 1.36	0.095
Osteosarcoma	1.09	0.48, 2.47	0.840
Rhabdomyosarcoma	2.00	0.92, 4.37	0.082
Synovial Sarcoma	0.60	0.25, 1.48	0.270
Stage			
Localized	–	–	
Metastatic	1.48	0.98, 2.25	0.063

*adj. SHR* adjusted subdistribution hazard ratio, Fine and Gray.

## Discussion

Herein we present a comprehensive analysis of BrM incidence and associated risk factors in 5110 sarcoma patients who presented over greater than 35 years to a large tertiary center. Of note, 53 patients were diagnosed and treated before presenting to our center. Four of them developed BrM: 3 had ASPS and 1 had fibrosarcoma. Compared to previous studies reporting BrM incidences ranging from 0.7% to 8%, our longitudinal analysis revealed CuI rates of 1.8%, 2.4%, and 2.9% at 1, 4, and 6 years, respectively [1–4, 7]. Chou et al. reported a CuI of 3.9% at 5 years and 8.4% at 10 years among 611 sarcoma patients, noting unique latency discrepancies between STS and bone sarcomas [8]. In that report, the risk for BrM in STS patients continued to rise over the entire length of follow up, whereas it plateaued after 3 years for bone sarcoma [8]. Our findings align with that: median TTBrM was 17 months (range 0–335 months) across our entire cohort; for bone sarcomas, it was 16.5 months (range 0–36 months), and no BrM were observed after 3 years following initial diagnosis (Fig. 3). Our cohort’s median TTBrM falls in the range of prior series (12–26.4 months) [2, 9, 10]. Remarkably, however, in the STS cohort, we continued to observe BrM cases up to 27 years after diagnosis (Fig. 3), underscoring the long-term metastatic risk in this group.

Four patients developed BrM 14 years after the initial primary sarcoma diagnosis. Interestingly, among those, 3 had ASPS, and 1 was fibrosarcoma, with no otherwise discernible commonalities. Within our cohort, 20 patients had ASPS, 7 of whom developed BrM, with median TTBrM of 96 months (range, 0–335); highlighting the propensity of ASPS for BrM, including latently. This is concordant with a previous report where ASPS tended to metastasize to the brain late in their disease course with a median TTBrM of 55 months [9]. Our study documents the longest reported TTBrM that we are aware of: 27 years (biopsy-proven).

Risk factors associated with BrM in sarcoma patients have not been well characterized. A SEER analysis conducted on 26,676 patients, that identified patients with BrM at presentation only, revealed a higher incidence of BrM in deeply located, higher grade and truncal tumors [6]. In addition, ASPS, malignant hemangioendothelioma and malignant schwannoma histologies had the highest rates of BrM incidence [6]. Nonetheless, this analysis only examined BrM incidence at presentation. Herein, we found higher CuI of BrM in patients with intrathoracic primary locations, ASPS histological subtypes and younger age at primary diagnosis. In line with previous studies that identify ASPS as having the highest BrM incidence (16–43.4%), our analysis revealed rates of: 10.8%, 17.2%, and 36.8% at 2, 6 and 27 years following diagnosis [1, 11]. Notably, NCCN guidelines already recommend central nervous system (CNS) imaging in ASPS and angiosarcoma, aligning with our findings [12].

Unique to this report, on univariable analysis, we noted that epithelioid sarcoma also exhibited a high incidence of BrM (8.4%). Epithelioid sarcoma, first described in 1970, has proximal and distal variants and is rare, accounting for <1% of all soft tissue sarcomas. It is characterized by high rates of local recurrence (25%) and distant relapse (30–50%) [13]. To our knowledge, this is the first study to describe high incidence of BrM in epithelioid sarcoma. While intimal sarcoma was not associated with a higher CuI of BrM on multivariable analysis, 13 patients with intimal sarcoma were identified in our cohort and 3 (23%) developed BrM with TTBrM of 5-8 months. This illustrates a high incidence of BrM early in the course of disease. Liposarcoma was associated with a lower risk of BrM (HR = 0.11), with an incidence of 0.27%, lower than previously reported (1.5–15%). Out of 143 patients with myxoid liposarcoma, one developed BrM. Of 180 cases of chondrosarcoma, none developed BrM. Finally, out of 29 patients with myxoid chondrosarcoma, 1 developed BrM, consistent with the studies reporting an incidence of (0–2%) [1, 14]. Our report is the first to demonstrate that intrathoracic primaries are associated with a higher incidence of BrM. The higher probability of intrathoracic primary may be attributed to the fact that most are either of cardiac or great vessel origin which could hypothetically result in seeding of metastatic emboli to the brain.

Compared to median age of 55 years reported in a SEER analysis (BrM at presentation only), our BrM cohort had a younger median age at diagnosis (47 years). Note that our cohort otherwise tended to be older than patients reported in prior studies (23 and 29.5 years) because we exclusively enrolled adults [9, 10].

Median survival in our cohort following BrM diagnosis was 6 months, which is relatively short compared to modern clinical trials that have enrolled BrM patients with primarily lung and breast cancer [15, 16]. Among histological subtypes, ASPS showed the highest median survival (53 months) while Endometrial Stromal Sarcoma (ESS), Malignant Peripheral Nerve Sheath Tumor (MPNST) and Rhabdomyosarcoma (RMS) showed the lowest, 0, 2 and 3 months, respectively. This is consistent with previous studies where median survival ranged from 2 to 7 months [17]. That said, we did not find statistically significant differences in median survival according to tested variables. Nguyen et al., recently published a systematic review and meta-analysis evaluating the impact of several variables (gender, number of BrM, surgery and the presence of lung metastases) on mortality among sarcoma BrM, however, none of these variables were associated with survival [17]. In our analysis, survival appeared to differ by treatment modality: patients treated with surgery followed by SRS had the most favorable outcomes, a median survival of 14 months, while WBRT alone was associated with the poorest (median 2 months). These differences likely reflect both selection factors and potential therapeutic impact, highlighting the importance of individualized, multidisciplinary management strategies.

We report a higher rate of hemorrhagic (39.7%) BrM than previously reported (30%) [9]. There was no difference in the median survival of patients with or without hemorrhagic BrM. Among patients who had hemorrahgic BrM, the most common subtypes were UPS (9/46, 19.5%), angiosarcoma (4/46, 8.7%) and ASPS, RMS and synovial sarcoma (3/46, 6.5%) each. Ten patients were found to have leptomeningeal disease (LMD), and interestingly, 4 had RMS histology. While 10% LMD rate was observed, it was lower than anticipated given the 40% hemorrhagic metastasis rate. Further investigation is needed to assess potential contributing factors such as treatment modalities.

Although our study provides some novel findings, it has several limitations. It is retrospective and was conducted at a single institution. The inclusion of 53 patients diagnosed before 2006 introduces potential selection bias because their diagnostic and treatment data may not align with the post-2006 cohort due to advancements in diagnostic imaging, therapeutic strategies, and follow-up protocols during the study period. However, we opted to retain these patients in our analysis since 4 developed BrM, including 3 from ASPS since their inclusion highlighted the potential for prolonged latency associated with ASPS. In addition, the BrM incidence was identified primarily through our center’s radiation records. Therefore, patients who did not undergo radiotherapy at our center would not have been captured; we expect but cannot prove that this is likely to have been a very small number of individuals. Diagnosis of BrM was based primarily on imaging (MRI), with histologic confirmation available in surgically treated cases (one third of BrM cohort); data on germline cancer predisposition and secondary malignancies were limited and not systematically analyzed. Finally, and perhaps most importantly, sarcoma patients are not regularly screened with brain MRIs, either at diagnosis or during routine follow up; the incidence we report should therefore be understood to reflect disease that was discovered in most instances because of patient symptoms that led to brain imaging.

In conclusion, we provide a comprehensive detailed analysis of a large cohort across a high-volume cancer center describing the CuI of BrM in sarcoma patients. While routine brain MRI for all sarcoma patients is not feasible due to low incidence, our findings may help identify high-risk groups who warrant further evaluation. Furthermore, the integration of machine learning and artificial intelligence offers a promising avenue to develop models that could predict the incidence of BrM among sarcoma patients. Our results underscore the heterogeneity of sarcoma and its metastatic behavior and raise the possibility of tailored surveillance protocols based on histological subtype and location of the primary tumor to identify BrM more effectively at the earliest possible stage to improve patients’ outcomes.

## Data Availability

The data are available from the corresponding author upon reasonable request.
